# KIF2C Is a Novel Prognostic Biomarker and Correlated with Immune Infiltration in Endometrial Cancer

**DOI:** 10.1155/2021/1434856

**Published:** 2021-10-05

**Authors:** Lanfen An, Jun Zhang, Dilu Feng, Yingchao Zhao, Weixiang Ouyang, Rui Shi, Xing Zhou, Zhicheng Yu, Sitian Wei, Jie Min, Hongbo Wang

**Affiliations:** ^1^Department of Obstetrics and Gynecology, Union Hospital, Tongji Medical College, Huazhong University of Science and Technology, Wuhan 430022, China; ^2^Cancer Center, Union Hospital, Tongji Medical College, Huazhong University of Science and Technology, Wuhan 430022, China

## Abstract

Endometrial cancer (EC) is commonly diagnosed cancer in women, and the prognosis of advanced types of EC is extremely poor. Kinesin family member 2C (KIF2C) has been reported as an oncogene in cancers. However, its pathophysiological roles and the correlation with tumor-infiltrating lymphocytes in EC remain unclear. The mRNA and protein levels of KIF2C in EC tissues were detected by qRT-PCR, Western blot (WB), and IHC. CCK8, Transwell, and colony formation assay were applied to assess the effects of KIF2C on cell proliferation, migration, and invasion. Cell apoptosis and cell cycle were analyzed by flow cytometry. The antitumor effect was further validated in the nude mouse xenograft cancer model and humanized mouse model. KIF2C expression was higher in EC. Knockdown of KIF2C prolonged the G1 phases and inhibited EC cell proliferation, migration, and invasion in vitro. Bioinformatics analysis indicated that KIF2C is negatively correlated with the infiltration level of CD8^+^ T cells but positively with the poor prognosis of EC patients. The apoptosis of CD8^+^ T cell was inhibited after the knockdown of KIF2C and was further inhibited when it is combined with anti-PD1. Conversely, compared to the knockdown of KIF2C expression alone, the combination of anti-PD1 further promoted the apoptosis of Ishikawa and RL95-2 cells. Moreover, the knockdown of KIF2C inhibited the expression of Ki-67 and the growth of tumors in the nude mouse xenograft cancer model. Our study found that the antitumor efficacy was further evaluated by the combination of anti-PD1 and KIF2C knockdown in a humanized mouse model. This study indicated that KIF2C is a novel prognostic biomarker that determines cancer progression and also a target for the therapy of EC and correlated with tumor immune cells infiltration in EC.

## 1. Introduction

Endometrial cancer (EC) is the sixth most commonly diagnosed cancer in women, which threatens women's health and quality of life, increasing the disease risk and medical burden on society. It was estimated that there are 417,000 new cases and 97,000 deaths in 2020 [1]. In China, the EC incidence has an upward tendency [2]. Most patients are diagnosed at an early stage with a good prognosis. However, the prognosis of advanced, poorly differentiated, or special types of EC is extremely poor [1, 3]. Therefore, exploring the pathogenesis and effective treatment targets of EC is of paramount importance to improve the prognosis of EC.

Kinesins are the microtubule-associated motor that produces mechanical work correlated with ATP hydrolysis [4]. Kinesin-13 is the branch of the kinesin superfamily of proteins and is the important regulator of microtubule dynamics during mitosis [5]. Kinesin family member 2A, kinesin family member 2B, and kinesin family member 2C (KIF2C) are three distinct genes encoding a member of kinesin-13, and KIF2C is the best-characterized member which located in the cytoplasm throughout the cell cycle [6, 7]. KIF2C is important for mitosis, participating in kinetochore-microtubule attachment, spindle assembly, chromosome congression, and segregation [8, 9]. Increasing microtubule KIF2C protein levels increased chromosome instability [10]. Additionally, KIF2C has been identified in cytoskeletal remodeling during tumor metastasis [11, 12]. KIF2C has also been reported as a putative oncogene that is highly expressed in hepatocellular carcinoma non-small-cell lung cancer, colorectal cancer, glioma, and gastric cancer [13–17]. Recently, increasing evidence indicated that KIF2C is associated with the prognosis of EC [18, 19]. However, its pathophysiological roles in EC remain uninvestigated.

The immune system is the core defense against cancer development and progression [20]. Tumor immunotherapies have triggered a paradigm shift in cancer therapy and have shown sustained clinical responses [21]. Currently, the most effective immunotherapies are immune checkpoint blocking (ICB) antibodies, which target inhibitory surface receptors expressed by T cells, particularly, programmed cell death receptor-1 (PD-1) [22]. Activated CD8^+^ T and CD4^+^ T cells expressed PD-1, and exhausted CD8^+^ T cells expressed PD-1 highly but showed diminished cytotoxic responding to antigens. In recent years, the importance of CD8^+^ T cells and the response to immunotherapy were studied [23]. A prior study reported that liver metastasis induced tumor-specific CD8^+^ T cell loss in preclinical models, which mirror the systemic CD8^+^ T cell loss and reduced immunotherapy efficacy observed in patients with liver metastasis [24]. High CD8^+^ T cells immune infiltration is found to be correlated with a good prognosis in melanoma [25]. The high density of direct CD8^+^ T/B cell interactions also can predict patients with an excellent prognosis, who would receive less invasive treatment in oropharyngeal squamous cell carcinoma [26]. Pretreatment peripheral CD8^+^ T cell levels are associated with checkpoint blockade response [27]. Conversely, disfunction or less infiltration of CD8^+^ T cells in the TME also results in worse clinical outcomes in many other cancer therapies [28–30]. Therefore, promoting the function and infiltration of CD8^+^ T cells in the TME may contribute to the efficacy of cancer therapies.

NY-CO-58/KIF2C has been revealed as a tumor antigen by screening antibodies in colorectal cancer [31]. However, the role of KIF2C in EC and its potential mechanisms in influencing CD8^+^ T cell antitumor efficacy have not been explored.

Here, we investigated the expression of KIF2C in EC, which is correlated with the progress and prognosis of EC. We also blockade the PD1/PD-L1 pathway to inhibit the CD8^+^ T cells immunology in vitro and in vivo. The findings of our study indicate that KIF2C influences the prognosis of EC and as a target for the therapy of EC and correlated with tumor immune cell infiltration in EC.

## 2. Materials and Methods

This study was conducted following the Declaration of Helsinki (as revised in 2013).

### 2.1. Data Collection

Level 3 expression data and mRNA expression profiles (575 cases, including 23 normal cases, workflow type: HTseq-FPKM) were downloaded from The Cancer Genome Atlas (TCGA) database. Clinical characteristics regarding survival time for EC patients were also obtained from the TCGA and Gene Expression Omnibus (GEO) database. R software (version 3.5.2) or Practical Extraction and Report Language (Perl) scripts [32] on JAVA software were used to process all data.

### 2.2. Gene Set Enrichment Analysis (GSEA)

To explore the potential correlations underlying the effect of KIF2C expression on EC prognosis, we applied GSEA (v4.1.0) to identify biological pathways pertaining to EC pathogenesis-associated KIF2C regulatory networks [33]. KIF2C-high expression and KIF2C-low expression were the phenotype labels. Each analysis of gene set permutations was conducted 1000 times.

### 2.3. Cibersort

The “limma” package [34] was used to normalize transcriptome profiles of EC patients. Next, we performed the “CIBERSORT” scripts to assess the immune compositions of EC patients [35]. We considered that the selected samples were usable when *p* < 0.05.

### 2.4. Patients' Sample

Twelve EC tissues and twelve adjacent tissues were collected from patients with who had undergone surgical resections or biopsies at the Department of Gynecology, Wuhan Union Hospital, from September 2019 to March 2021. Complete clinical data were available for all patients. At least two pathologists evaluated all specimens according to the World Health Organization (WHO) guidelines. This study was approved by The Ethics Committees of Union Hospital, Tongji Medical College, Huazhong University of Science and Technology. We obtained patients' permission before surgery. Tissues are placed in 10% formalin immediately and embedded in paraffin for future use. The rest part of the tissue samples was immediately aliquoted frozen and stored in liquid nitrogen for further RNA and protein extraction.

### 2.5. Cell Culture

Human EC cell lines Ishikawa and RL95-2 were obtained from American Type Culture Collection (ATCC, Manassas, USA). Ishikawa cells were cultured in DMEM/F12 (Gibco, Thermo Fisher Scientific, Massachusetts/MA, USA) with 10% fetal bovine serum (FBS, Biological Industries, Beit HaEmek, Israel) and 1% penicillin and streptomycin (PS, Boster, Wuhan, China) at 37°C under a humidified atmosphere of 5% CO_2_. RL95-2 cells were cultured in DMEM/F12 supplemented with 10% FBS, 1% PS, and 1% insulin (Novo Nordisk, Copenhagen, Denmark) at 5% CO_2_ and 37°C.

### 2.6. Transfection

Lentivirus containing KIF2C-shRNA and shNC was also purchased from GenePharma (Shanghai, China). The effective KIF2C-shRNA sequences were 5′-CCAACGCAGUAAUGGUUUATT-3′. Seventy-two hours after transfection, WB was used to examine the KIF2C-shRNA interference effect. Stable cells transfected with KIF2C-shRNA were obtained after 14 days of puromycin screening (2 *μ*g/ml).

### 2.7. Reverse Transcription and Quantitative Real-Time Polymerase Chain Reaction (qRT-PCR) Analysis

Total RNA was isolated samples of cells and EC samples using RNAiso Plus (TaKaRa, Shiga, Japan) following manufacturer's protocol; cDNA was prepared using ABScript RT Master Mix (TaKaRa, Shiga, Japan). qRT-PCR was performed using the CFX Connect Real-Time System (Bio-Rad, California, USA) with primers specific for KIF2C, forward 5′-TCCAGGCAATTTATCCAAGG-3′, reverse 5′-CCAGTCTGGTCCTTGCTGTA-3′; GAPDH, forward 5′-AGATCCCTCCAAAATCAAGTGG-3′, reverse 5′-GGCAGAGATGATGACCCTTTT-3′. Target gene expression was quantified relative to the expression of GAPDH using the comparative Ct (2^−ΔΔCt^) method.

### 2.8. WB Analysis and Antibodies

WB assay was implemented as previously described [36]. The primary antibodies are against KIF2C (1 : 1000 dilution, Proteintech, Wuhan, China) and GAPDH (1 : 10000 dilution, ABclonal, Woburn, MA, USA). After incubation with peroxidase-labeled species-specific secondary antibodies (1 : 8000 dilution, ABclonal, Woburn, MA, USA), protein bands were visualized by ECL substrate (Servicebio, Wuhan, China) using Image Lab Software in Molecular Imager ® ChemiDoc™ XRS+ (Bio-Rad, California, USA).

### 2.9. Immunohistochemistry (IHC) Staining and Scoring

The EC tissues embedded in paraffin were sectioned into slices 4 *μ*m thick, and the slides were then deparaffinized and rehydrated. After being treated with 3% hydrogen peroxide, the slides were incubated overnight at 4°C with the primary antibody KIF2C (1 : 500 dilution, Proteintech, Wuhan, China). After rinsing in PBS, the sections were incubated with peroxidase-labeled anti-rabbit immunoglobulin G (IgG) for 30 min. Finally, all slides were incubated with DAB-Substrate (Beyotime, Shanghai, China) before being rinsed with distilled water, counterstained with haematoxylin, dehydrated, and mounted. A Motic microscope (Motic, Xiamen, China) was used to visualize and photograph the slides. Semiquantitative evaluation of IHC was performed by two observers who were blinded to the identity of the slides. Remmele and Schicketanz immunoreactive score (IRS) and immunohistochemical scores (IHS) were selected to analyze the data we obtained [37]. The multiplex fluorescent IHC staining intensity was obtained by image J. The IHS was determined based on the staining intensity (SI) and the percentage of immunoreactive cells (PR). The SI scale was divided into four categories (0 = no staining, 1 = weak staining, 2 = moderate staining, and 3 = strong staining), and the PR scale was divided into five categories (0 = no staining, 1 = 1-10% staining, 2 = 11-50% staining, 3 =51-80% staining, and 4 = 81-100% staining). A final semiquantitative score ranging from 0 to 12 points was calculated for each sample. Samples with IHS above 4 were considered positive, and those with IHS below 4 were considered negative [37].

### 2.10. Cell Proliferation Assay

Ishikawa and RL95-2 cells were seeded in 96-well plates (Nest Biotechnology, Wuxi, China) at the density of 2 × 10^3^ and 4 × 10^3^ per well, respectively. Cell-Counting Kit-8 (CCK8, Bimake, Houston, USA) was used to determine the cell proliferation following the user manual. The resulting absorbance at 450 nm was recorded using a spectrophotometer (ThermoFisher Scientific, Waltham, MA, USA). All CCK8 assays were conducted in triplicate.

### 2.11. Cell Migration and Invasion Assays

Transwell chambers of 8 *μ*m pore size (Corning Costar, Maine/ME, USA) were used to assess migration and invasion. Serum-free medium was used in the top chamber with Ishikawa (8 × 10^4^/well) and RL95-2 (1.2 × 10^5^/well), respectively. The microfilters were precoated with 50 *μ*l of Matrigel matrix (BD Biosciences, Sparks, MD, USA) in invasion assays. The bottom chambers contained a chemo-attractant (medium with 10% FBS). After 24-hour migration and invasion, the cells were then fixated and stained with 0.1% violet crystal (Servicebio, Wuhan, China). Cells that passed the membrane were counted and imaged in 3 random fields of the membrane using CX23 Olympus light microscopy (Olympus, Tokyo, Japan).

### 2.12. Colony Formation Assay

Ishikawa and RL95-2 cells were seeded into 8 cm^2^ dishes at a density of 700 and 1000 cells and then allowed to grow for 2 weeks. Cells were fixed with methanol (Servicebio, Wuhan, China) for 15 min and stained with 0.1% violet crystal (Servicebio, Wuhan, China) for 30 min and counted manually.

### 2.13. Flow Cytometry

After transfection of the shRNA and shNC for KIF2C, Ishikawa and RL95-2 cells (1 × 10^6^) were collected with EDTA (Gibco, ThermoFisher Scientific, Waltham, MA, USA) and washed three times with phosphate-buffered saline (PBS, Servicebio, Wuhan, China) and fixed on ice with 70% ethanol. Flow cytometry was implemented as previously described [36].

### 2.14. Human-Activated CD8^+^ T Cell Collection

Using a human lymphocyte separation medium (Dakewe, Shenzhen, China) isolated healthy human donor peripheral blood mononuclear cells (PBMCs). CD8^+^ T cells were isolated from PBMCs by magnetic bead purification using human CD8^+^ T cell microbeads (Miltenyi Biotec, Bergisch Gladbach, Germany). Then, the CD8^+^ T cells were cultured in Roswell Park Memorial Institute- (RPMI-) 1640 medium with 10% fetal bovine serum (Gibco, Thermo Fisher Scientific, MA, USA) and 1% PS (Boster, Wuhan, China). Dynadeads™ human T-activator CD3/CD28 (Thermo Fisher Scientific, MA, USA) was used to CD8^+^ T cell expansion and activation [38].

### 2.15. In Vitro CD8^+^ T Cell Coculture System

In a 3-day incubation, magnetic bead-purified peripheral CD8^+^ T cells (2 × 10^5^ cells/well in 21 cm^2^ plates) were cocultured with Ishikawa and RL95-2 cells with shKIF2C and shNC at a 2 : 1 (CD8^+^ T cell : tumor cell) ratio in 5 ml complete RPMI-1640 medium, in the presence or absence of human monoclonal antibody PD-1 neutralizing antibody camrelizumab (SHR1210) (20 *μ*g/ml, Heng Rui, Jiang Su, China) [39]. After 3-day incubation, the supernatants were harvested for LDH assay, and the CD8^+^ T cells were harvested for analysis of CD8^+^ T cells apoptosis. The proportion of apoptosis CD8^+^ T cell was examined using PE annexin V apoptosis detection kit (BD Biosciences, New Jersey, USA). Flow cytometry was performed using the FACSVerse flow cytometer (BD Biosciences, New Jersey, USA), and the data were analyzed with FlowJo software (TreeStar, USA).

### 2.16. Lactate Dehydrogenase (LDH) Assay

LDH is a cytoplasmic enzyme in cells and releases rapidly after the plasma membrane is damaged. Thus, LDH is used as an indicator of necrotic cell death [40]. CD8^+^ T cells cocultured with Ishikawa and RL95-2 cells with shKIF2C and shNC at a 2 : 1 (CD8^+^ T cell : tumor cell) ratio; after a 3-day incubation, the cell culture supernatant was collected, and the level of LDH was detected by LDH cytotoxicity assay detection kit (Beyotime, Shanghai, China) according to manufacturer's protocol.

### 2.17. Nude Mouse Xenograft Cancer Model

All animal studies were performed according to the protocols approved by Tongji Medical College's Animal Care and Use Committee. BALB/c-nu nude mice were purchased from China Beijing Vital River and housed in a standard pathogen-free environment laboratory. The mice were randomized into two groups. Xenografts were initiated by subcutaneous injection of total 1 × 10^6^ negative or KIF2C-silenced Ishikawa cells in 100 *μ*l PBS into the right flank of Bulb/c nude mice. After 15 days, the mice were euthanized, and the weight of xenografts was recorded. Tumor volumes were measured using the formula (length × width^2^)/2. Tumor samples were partially embedded in paraffin for histopathological analysis.

To test the suppressive effect of PD-L1^+^ EC cells on CD8^+^ T cells immunity in vivo, we treated the mice with a subcutaneous injection of a total of 5 × 10^6^ Ishikawa cells and with intravenous injection activated CD8^+^ T cells (2.0 × 10^6^) from human PBMCs [41]. For in vivo checkpoint blockade, 5-week mice were administrated with 250 *μ*g PD1 monoclonal antibody through intraperitoneal injection (IP) three times [42].

### 2.18. Statistical Analysis

The correlations between the expression of KIF2C and clinical features were evaluated by the logistic regression and Wilcoxon signed-rank test. The KIF2C expression of the EC cohort was obtained using box plots. Clinical factors associated with OS in EC were identified using the Cox regression and Kaplan-Meier method. Quantitative values are expressed as the means ± SEM. Student's *t*-test or one-way ANOVA was used to compare multiple groups. Correlation analysis was performed by the Spearman rank test. Statistical significance was defined as *p* value < 0.05.

## 3. Results

### 3.1. KIF2C Is Highly Expressed in EC and Correlated with the Expression of CD8^+^ T Cells

**Figure 1:**
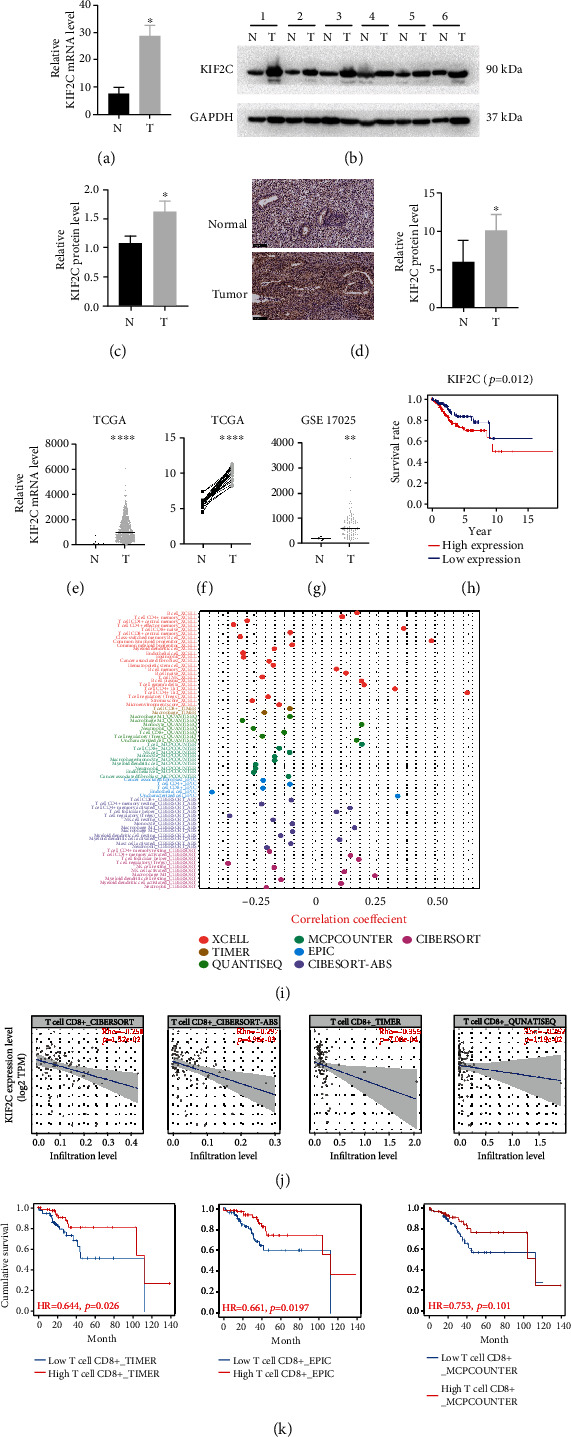
KIF2C is highly expressed in human EC and correlated with the level of immune infiltration. (a–d) The KIF2C mRNA and protein level was upregulated in fresh EC tissue, and determined by qRT-PCR (a), WB (b, c), and IHC (d). (e) Transcriptional level of KIF2C in EC (tumor) and benign endometrium (normal) tissues from the TCGA. (f) Upregulation of KIF2C mRNA level in EC of twenty-three paired samples from the TCGA. (g) KIF2C mRNA expression mined from GEO databases (GSE17025). (h) Kaplan-Meier survival curves compare high and low expression of KIF2C for survival rate in EC. (i) Correlation analysis between immune cells and EC in TCGA. (j) KIF2C expression is correlated with the CD8^+^ T cell infiltration level in EC. (k) Kaplan-Meier curves compare low and high expression of CD8^+^ T cell infiltration level for cumulative survival in EC. T: endometrial cancer tissue; N: normal endometrium; error bars mean ± SEM. ^∗^*p* < 0.05; ^∗∗^*p* < 0.01; ^∗∗∗^*p* < 0.001; ^∗∗∗∗^*p* < 0.0001.

In this study, we determined the KIF2C mRNA expression by qRT-PCR (Figure 1(a)). Using WB, we analyzed the expression level of KIF2C in 12 cases of EC tissue and 12 normal endometrium cases (Figures 1(b) and 1(c)). As shown, KIF2C was located in the cytoplasm according to IHC (Figure 1(d)). Moreover, these results showed that the KIF2C expression level was significantly higher in EC samples compared with normal endometrium samples. To elucidate the expression of KIF2C and the significance of upregulation of KIF2C in EC, we mined the publicly available databases Gene Expression Omnibus website (GEO; https://www.ncbi.nlm.nih.gov/geo/) and TCGA. The correlation of KIF2C expression with patients' clinicopathological features in primary EC was shown in Table 1. We analyzed the microarray dataset from TCGA containing 552 tumor samples and 23 normal samples. KIF2C expression was higher in EC samples than in control group samples (Figure 1(e)). We then analyzed 23 paired samples from TCGA and GSE17025 microarray datasets from GEO; also, the differential expression results were consistent with the overall results (Figures 1(f) and 1(g)). TCGA dataset analysis revealed that high-level expression of KIF2C is correlated with older age, vital status, higher pathologic grade, and higher clinical stage (Supplementary Figures 1(a)–1(d)). There are no significant differences between KIF2C expression and survival time (Supplementary Figure 1(e)), but Kaplan-Meier analysis showed that higher KIF2C expression was significantly associated with a low survival rate in the TCGA cohort (Figure 1(h)).

To analyze the relationship between the expression of KIF2C and immune-cell characteristics, we considered the current acknowledged methods to calculate the immune infiltration status among the samples from TCGA project of the uterine corpus endometrial carcinoma (UCEC) dataset including TIMER, XCELL, QUANTISEQ, MCPCOUNTER, EPIC, CIBERSORT-ABS, and CIBERSORT. The UCEC patients were divided into two groups according to the KIF2C expression level (high vs. low) based on the median expression values across all samples. Our results indicated that CD8^+^ T cell expression was negatively correlated with EC. Details about the subsets of infiltrating immune cells are shown in Figure 1(i). Hence, we investigated the relationship between KIF2C and the CD8^+^ T cell. KIF2C expression was negatively correlated with the levels of CD8^+^ T cell (Figure 1(j)). Similarly, the CD8^+^ T cell expression level is positively correlated with the cumulative survival of EC (Figure 1(k)), which suggested that KIF2C may be involved in remodeling the tumor immune environment and thereby promoting the malignant progression of EC.

### 3.2. KIF2C Promotes Cell Proliferation, Migration, and Invasion In Vitro

**Figure 2:**
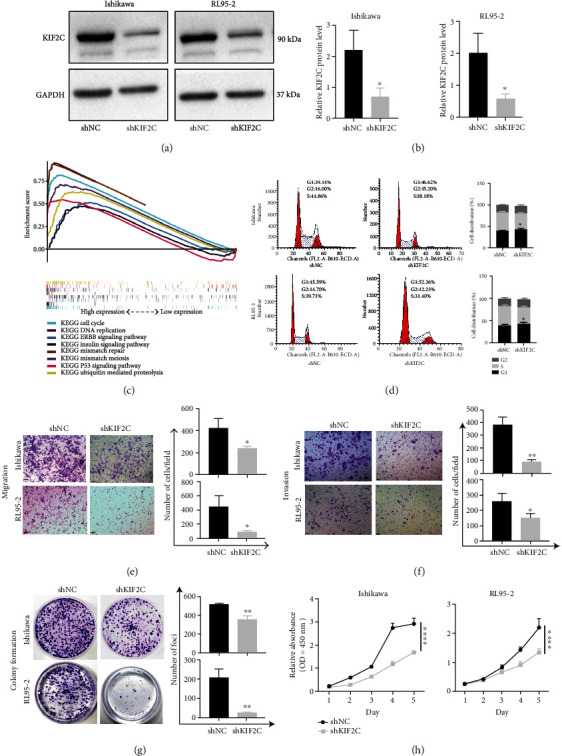
KIF2C expression is correlated with poor progress and outcomes in EC from the TCGA. (a, b) Knockdown the expression of KIF2C in Ishikawa and RL95-2 cells. (c) GSEA analysis suggests that KIF2C may be involved in some common signaling pathways, such as cell cycle, DNA replication, ERBB signaling pathway, oocyte meiosis, and ubiquitin-mediated proteolysis. (d) The cell cycle in Ishikawa and RL95-2 cells were analyzed by flow cytometry. (e–f) After being transfected with shNC and shKIF2C, the migration and invasion abilities of Ishikawa and RL95-2 cells were assessed by Transwell assay (*n* = 3). (g) The knockdown of KIF2C reduces the number of colonies in Ishikawa and RL95-2 cells (original magnification at ×100; error bars mean ± SEM; *n* = 3). (h) CCK8 assays were performed to determine the effect of shKIF2C on cell growth in vitro. Scale bars, 200 *μ*m; error bars mean ± SEM; ^∗^*p* < 0.05; ^∗∗^*p* < 0.01; ^∗∗∗^*p* < 0.001.

The increased transcriptional level of KIF2C was correlated with aggressive tumor behavior in EC, which reveals that KIF2C may play an essential role in most EC tumor development. To validate this hypothesis, loss-of-function studies for KIF2C were performed in EC cell lines. We stably transfected with shKIF2C in Ishikawa and RL95-2 cells, leading to a decreased protein level of KIF2C compared to those in cells transfected with shNC (Figures 2(a) and 2(b)). We divided 552 TCGA UCEC samples into two groups (high vs. low) according to the median values of expression levels and performed GSEA to investigate the biological pathways in which KIF2C may be involved based on the c2.cp.kegg.v7.0.symbols.gmt gene set. Finally, we obtained 26 common pathways at FDR < 0.25 and *p* < 0.05. We selected the representative pathways and plotted the GSEA diagram including the cell cycle, DNA replication, ERBB signaling pathway, insulin signaling pathway, mismatch repair, oocyte meiosis, ubiquitin-mediated proteolysis, and P53 signaling pathway (Figure 2(c)). Based on the analysis of GSEA, we found that KIF2C plays a pivotal role in the cell cycle. Subsequently, the potential roles of KIF2C in cell cycle regulation were validated. As shown in Figure 2(d), the G1 phases were significantly prolonged when KIF2C expression was reduced, which indicated that the knockdown of KIF2C could promote cell cycle arrest by restricting cells from entering the G2 phase.

Furthermore, Transwell and CCK8 assays were used to investigate cell migration, invasion, and proliferation. The Transwell assay revealed that the abilities of migration and invasion in Ishikawa and RL95-2 cells were inhibited when KIF2C was knocked down (Figures 2(e) and 2(f)). The colony formation assay also showed that the numbers of colonies were significantly reduced in KIF2C knockdown cells compared with control cells (Figure 2(g)). The results of the CCK8 assay indicated that the low expression of KIF2C significantly inhibited the viability of EC cells (Figure 2(h)).

### 3.3. Anti-PD1 Immunotherapy Enhances the Response to Inhibit EC Cells In Vitro

**Figure 3:**
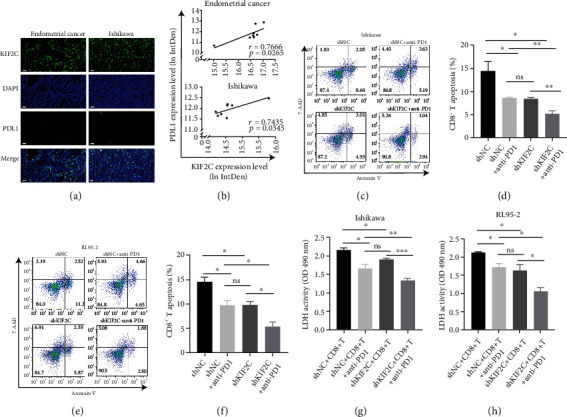
Anti-PD1 immunotherapy enhances the response to inhibit EC cells in vitro. (a) Representative images of KIF2C (green) and PDL1 (pink) in tumor tissues and Ishikawa cells were analyzed by multiplex fluorescent IHC (original magnification at ×200). (b) The correlations between KIF2C and PD-L1 in human EC and Ishikawa cells. Results were expressed as log (ln) of integrated density in EC tissues and Ishikawa cells. (c–f) After being transfected with shNC or shKIF2C, Ishikawa and RL95-2 were cocultured for 3 days with activated CD8^+^ T cells, in the presence or absence of anti-PD1. Representative data and statistical analysis of T cell apoptosis were shown (*n* = 3). (g, h) The LDH released in cell cocultured supernatant was measured at the indicated time (*n* = 3). Scale bars, 50 *μ*m; error bars mean ± SEM.

KIF2C overexpression was revealed to be correlated with reduced immune infiltration in lung adenocarcinoma [42]. Thus, we investigated the expression of KIF2C and PD-L1 in the EC tissues and Ishikawa cells. And we observed that the expression of PD-L1 is positively correlated with KIF2C (Figures 3(a) and 3(b)). To further validate whether upregulation of KIF2C contributed to the suppressive effects on CD8^+^ T cell immunity, Ishikawa and RL95-2 cells were stably transfected with shNC and shKIF2C and cocultured with in vitro-activated CD8^+^ T cells in 21 cm^2^ plates with or without anti-PD1. Then, CD8^+^ T cells were collected for flow cytometric analysis. Ishikawa transfected with shNC or shKIF2C cocultured with CD8^+^ T cells showed a higher percentage of apoptosis compared to that with CD8^+^ T cells and anti-PD1 antibody (Figures 3(c) and 3(d)). And the results of RL95-2 cells cocultured with CD8^+^ T cell and PD1 antibody are consistent with the results in Ishikawa cells (Figures 3(e) and 3(f)), which revealed that the reduced KIF2C expression inhibits the apoptosis of CD8^+^ T cells, and the anti-PD1 antibody further inhibits the apoptosis of CD8^+^ T cells.

Moreover, LDH is a stable cytoplasmic enzyme in all cells, which can release rapidly following plasma membrane damage. To demonstrate the apoptosis of coculture of tumor cells and CD8^+^ T cells, samples were collected by taking the supernatant of culture at the time of CD8^+^ T cells were collected, and an LDH assay was carried out. The results of the LDH assay were consistent with cytometric analysis (Figures 3(g) and 3(h)). Thus, KIF2C contributes to the immune suppression of CD8^+^ T cells in the progress of EC.

### 3.4. KIF2C Promotes the Growth of EC Cells In Vivo

**Figure 4:**
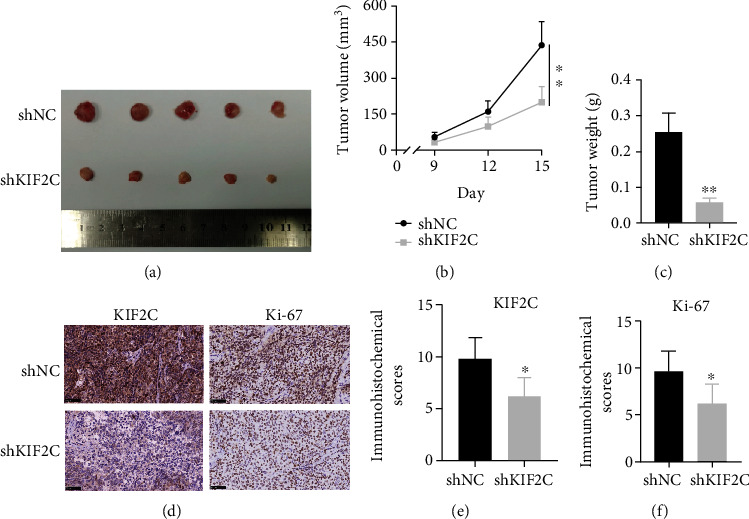
KIF2C promotes the growth of EC cells in vivo. (a) Representation xenograft tumors formed by injection of Ishikawa cells. (b, c) The volume and weight of the xenograft were measured. (d–f) The IHC analyses of KIF2C and ki-67 in xenograft tumors (original magnification at ×400). Scale bars, 50 *μ*m. Error bars mean ± SEM. ^∗^*p* < 0.05; ^∗∗^*p* < 0.01.

To investigate the role of the KIF2C in EC in vivo, we established a subcutaneous graft tumor model using Ishikawa cells in nude mice. Ishikawa cells stably transfected with shNC and shKIF2C were subcutaneously injected into nude mice. As expected, the implanted tumors generated from the shKIF2C group grew dramatically slower than those generated from shNC group (Figure 4(a)). The tumor volume and weight were reduced dramatically in the shKIF2C group (Figures 4(b) and 4(c)). The IHC showed that KIF2C, as well as Ki-67 protein expression, was evidently decreased in the shKIF2C group (Figures 4(d)–4(f)). These results revealed that KIF2C knockdown leads to EC cells growth restricted in vivo as displayed in vitro.

### 3.5. Blockade of PD1 on CD8^+^ T Cell Immunity Inhibits Tumor Growth In Vivo

**Figure 5:**
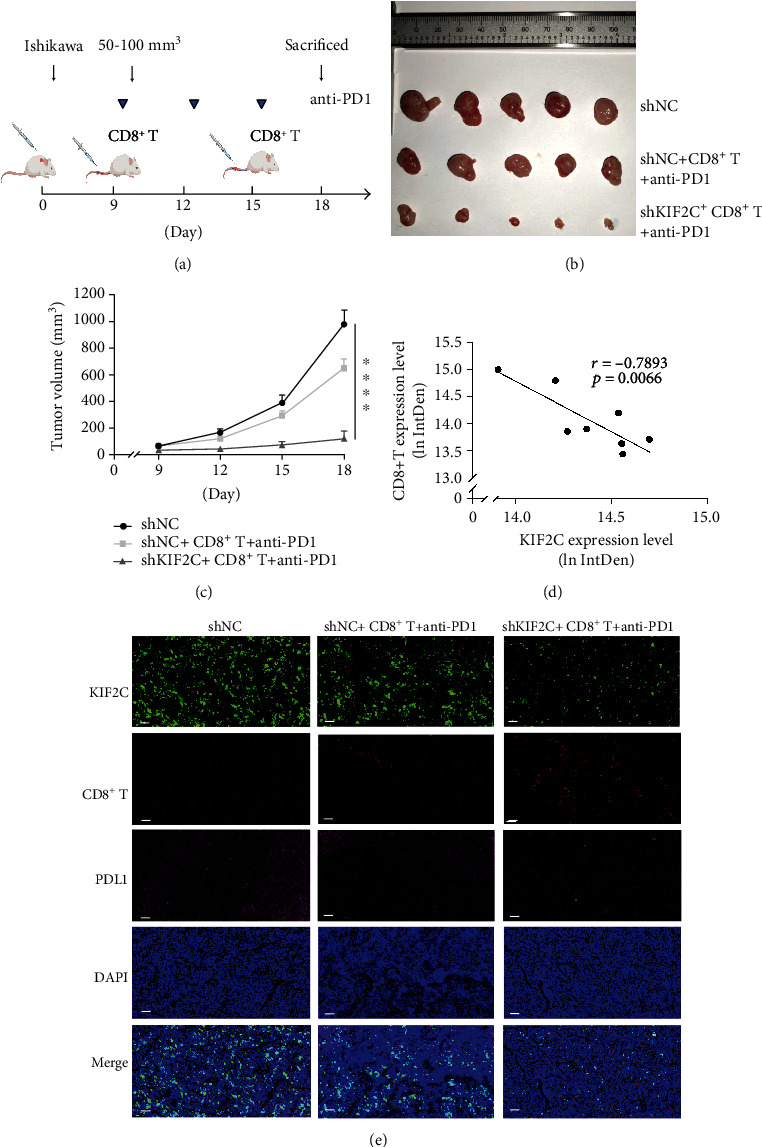
Blockade of PD1 on CD8^+^ T cell immunity inhibits tumor growth in vivo. (a) Mice were injected with human Ishikawa cells transfected with shKIF2C or shNC, the entailed injections with CD8^+^ T cell or CD8^+^ T cell in combination with an intraperitoneal injection of an anti-PD1 antibody. A schematic representation of the treatment was shown. (b, c) Representative images of tumors and a comparison of Ishikawa tumor growth in different groups were shown (*n* = 5 per group). (d) The correlation between KIF2C and CD8^+^ T cell in xenograft. Results were expressed as log (ln) of integrated density in xenograft. Each dot in (d) represents 1 field. (e) Representative analysis of KIF2C (green), DAPI (blue), CD8^+^ T cell (red), and PDL1 (pink) in xenograft by multiplex fluorescent immunohistochemistry (original magnification at ×200). Scale bars, 50 *μ*m. Error bars mean ± SEM. ^∗^*p* < 0.05; ^∗∗^*p* < 0.01; ^∗∗∗^*p* < 0.001; ^∗∗∗∗^*p* < 0.0001.

Because of the small size of the previously formed xenograft, we increased the number of inoculations when subcutaneous injection. To test the suppressive effect of PD1 on CD8^+^ T cell immunity in vivo, xenografts were initiated by subcutaneous injection of total 5 × 10^6^ Ishikawa cells in 100 *μ*l PBS into the right flank of Balb/c nude mice. We then asked whether the combination of CD8^+^ T cell and anti-PD1 antibody could potentiate the efficacy of EC cell growth in a xenograft mouse model. When the tumor volume reached 50-100 mm^3^, mice were treated with activated CD8^+^ T cell combination with anti-PD1 three times over 9 days (Figure 5(a)). There was a decrease in tumors in mice treated with CD8^+^ T cell and anti-PD1 compared with the control group. Mice treated with CD8^+^ T cell plus anti-PD1 antibody also showed decreased tumor proliferation (Figures 5(b) and 5(c)). We also observed that the KIF2C expression level is negatively correlated with CD8^+^ T cells in xenograft (Figure 5(d)). The representative images are shown in Figure 5(e).

## 4. Discussion

It is generally accepted that KIF2C has been reported to be upregulated in several cancers and promoted tumor proliferation and progression. In mammary carcinogenesis, overexpression of KIF2C might be involved in breast carcinogenesis and is a promising therapeutic target as well as a prognostic biomarker for breast cancer [43–45]. Recently, studies have revealed that KIF2C is overexpressed in hepatocellular carcinoma tissues. High KIF2C protein promotes cell proliferation and predicts poor prognosis [46]. Similarly, overexpression of KIF2C promotes bladder cancer progression [47]. However, the expression and role of KIF2C in EC have not been fully elucidated. This study demonstrated that KIF2C plays an essential role in EC progression.

Over the past decades, accumulated evidence has emphasized that the KIF2C plays a pivotal role in a variety of biological activities. For example, KIF2C has been revealed as a target of the mTORC1 and Wnt/*β*-catenin signaling in hepatocellular carcinoma [48]. KIF2C also exerts an oncogenic role and is negatively regulated by miR-325-3p in non-small-cell lung cancer (NSCLC) [13]. Jung et al. reported that KIF2C participates in the mechanism that lactate activates the E2F pathway to promote cell motility [49]. In breast cancer, comprehensive bioinformatics analyses revealed that tumor-related KIF2C correlated with poor outcomes of breast cancer patients and acts as a potential prognostic biomarker [45]. Here, we found that KIF2C was highly expressed in EC and associated with worse overall survival. This may be owing to the evidence that patients with high-level KIF2C expression have a higher advanced clinical stage, higher pathologic grade, and worse subtype. Employing loss-of-function studies, we demonstrated that decreasing KIF2C expression inhibited cell proliferation, migration, and invasion in vitro and inhibited tumor growth in vivo, suggesting an oncogenic role of KIF2C during EC progression. These results are consistent with a study that revealed that reduced KIF2C expression inhibits the migration and invasion of hepatocellular carcinoma [48].

To facilitate the elucidation of the biological function of the KIF2C in the development of EC, we performed GSEA. GSEA analysis showed that KIF2C was involved in the cell cycle. Using the flow cytometry assay, we confirmed that the knockdown of KIF2C could promote cell cycle arrest by restricting cells from entering the G2 phase, and this conclusion was consistent with those of a previously published study [50].

Anti-PD1 antibody enhances CD8^+^ T cell-mediated killing of tumor cells through removing CD8^+^ T cell suppressive signals from series of tumor-associated cells [51]. Li and Wan revealed the differences in immune phenotypes of the immune microenvironment in EC and defined four immune subtypes of EC, and CD8^+^ T cells were correlated with EC patients' survival [52]. A recent study investigated that quantification of intraepithelial CD8^+^ T cells also improves the prognostic utility of the molecular EC classification in the early stage [53].

In our study, we analyzed the correlation between KIF2C expression and the level of immune cell infiltration in EC tissues, and we found that KIF2C was inversely correlated with CD8^+^ T cells level. This finding suggested that the expression of KIF2C in EC may be correlated with CD8^+^ T cells. So, we investigated the potential mechanisms of KIF2C in influencing CD8^+^ T cells' antitumor efficacy in vivo and cytotoxicity in vitro. Intriguingly, CD8^+^ T cells cocultured with Ishikawa and RL95-2 showed a higher percentage of apoptosis compared to that with anti-PD1 antibody. This further supports our hypothesis that blockade of PD1 on CD8^+^ T cells immunity inhibits tumor growth in vitro and in vivo. However, the detailed mechanism needs to be further investigated.

In conclusion, our study sheds light on the biological and clinical significance of KIF2C in EC. Knockdown of KIF2C exerts a negative effect on cell proliferation, migration, and invasion. PD1 antibody blockade of immune receptors has proven to be effective in revitalizing exhausted CD8^+^ T cells. Targeting two or more receptors has a pivotal potential to increase the efficacy of immunotherapy. We find that the antitumor efficacy was further evaluated by the combination of anti-PD1 and KIF2C knockdown in a humanized mouse model. Our findings here suggest that KIF2C is a novel prognostic biomarker that determines cancer progression and also as a target for the therapy of EC, which correlated with tumor immune cells infiltration in EC.

## Figures and Tables

**Table 1:** Correlation of KIF2C expression with patients' clinicopathological features in primary endometrial cancer.

Parameter	KIF2C expression		*p* value
	Low	High	
Age	63 (56-71)	65 (57-72)	0.06
Clinical stage			<0.01
FIGO I	224	115	
FIGO II	25	26	
FIGO III	59	65	
FIGO IV	10	19	
Neoplasm histologic grade			<0.01
G1	92	6	
G2	98	22	
G3	128	197	
Histological type			<0.01
Endometrioid endometrial adenocarcinoma	277	130	
Mixed serous and endometrioid	10	12	
Serous endometrial adenocarcinoma	31	83	

## Data Availability

Data that are directly related to the acquired results are available. For more detailed information, the readers can consult Lanfen An (email: alf9105@163.com).

## Supplementary Materials

Supplementary Materials Correlation of KIF2C expression with patients' clinicopathological features in primary endometrial cancer (Figure s1).
