# Supplementary material for: KIF2C Is a Novel Prognostic Biomarker and Correlated with Immune Infiltration in Endometrial Cancer
Source: Stem Cells Int. 2021 Oct 5;2021:1434856. doi: 10.1155/2021/1434856 (PMC8510809; doi:10.1155/2021/1434856)
Supplement: Supplementary Materials — Correlation of KIF2C expression with patients' clinicopathological features in primary endometrial cancer (Figure s1). [file 1434856.f1.pdf]

Figure S1 Correlation of KIF2C expression with patients' clinicaopathological features in primary endometrial cancer

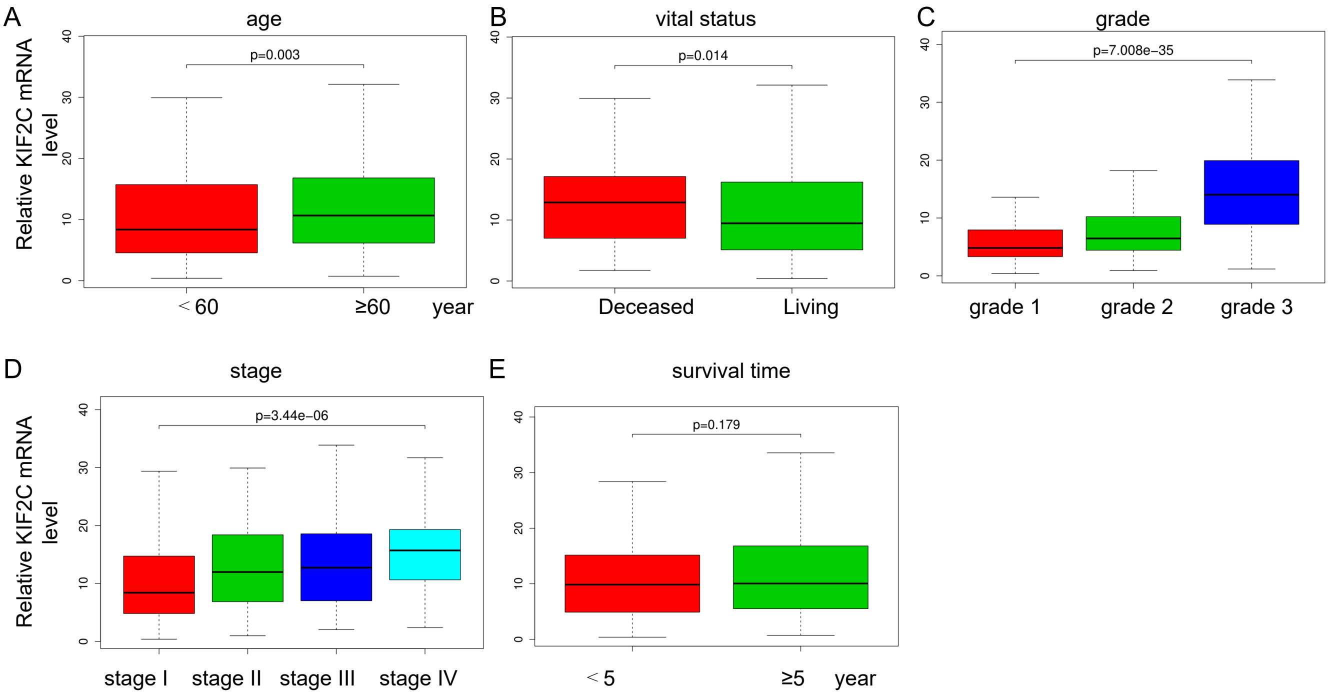

(A-E) The association between KIF2C expression and clinical characteristics of endometrial cancer patients in TCGA.
